# Scutellarin Suppresses NLRP3 Inflammasome Activation in Macrophages and Protects Mice against Bacterial Sepsis

**DOI:** 10.3389/fphar.2017.00975

**Published:** 2018-01-09

**Authors:** Yi Liu, Yan-Yun Jing, Chen-Ying Zeng, Chen-Guang Li, Li-Hui Xu, Liang Yan, Wen-Jing Bai, Qing-Bing Zha, Dong-Yun Ouyang, Xian-Hui He

**Affiliations:** ^1^Institute of Dermatology, Chinese Academy of Medical Sciences, Nanjing, China; ^2^Department of Immunobiology, College of Life Science and Technology, Jinan University, Guangzhou, China; ^3^Department of Cell Biology, College of Life Science and Technology, Jinan University, Guangzhou, China; ^4^Department of Fetal Medicine, The First Affiliated Hospital of Jinan University, Guangzhou, China

**Keywords:** scutellarin, NLRP3 inflammasome, pyroptosis, protein kinase A, macrophages

## Abstract

The NLRP3 inflammasome plays a critical role in mediating the innate immune defense against pathogenic infections, but aberrant activation of NLRP3 inflammasome has been linked to a variety of inflammatory diseases. Thus targeting the NLRP3 inflammasome represents a promising therapeutic for the treatment of such diseases. Scutellarin is a flavonoid isolated from *Erigeron breviscapus* (Vant.) Hand.-Mazz. and has been reported to exhibit potent anti-inflammatory activities, but the underlying mechanism is only partly understood. In this study, we aimed to investigate whether scutellarin could affect the activation of NLRP3 inflammasome in macrophages. The results showed that scutellarin dose-dependently reduced caspase-1 activation and decreased mature interleukin-1β (IL-1β) release in lipopolysaccharide (LPS)-primed macrophages upon ATP or nigericin stimulation, indicating that scutellarin inhibited NLRP3 inflammasome activation in macrophages. Consistent with this, scutellarin also suppressed pyroptotic cell death in LPS-primed macrophages treated with ATP or nigericin. ATP or nigericin-induced ASC speck formation and its oligomerization were blocked by scutellarin pre-treatment. Intriguingly, scutellarin augmented PKA-specific phosphorylation of NLRP3 in LPS-primed macrophages, which was completely blocked by selective PKA inhibitor H89, suggesting that PKA signaling had been involved in the action of scutellarin to suppress NLRP3 inflammasome activation. Supporting this, the inhibitory effect of scutellarin on NLRP3 inflammasome activation was completely counteracted by H89 or adenyl cyclase inhibitor MDL12330A. As NLRP3-dependent release of IL-1β has a critical role in sepsis, the *in vivo* activity of scutellarin was assayed in a mouse model of bacterial sepsis, which was established by intraperitoneally injection of a lethal dose of viable *Escherichia coli*. Oral administration of scutellarin significantly improved the survival of mice with bacterial sepsis. In line with this, scutellarin treatment significantly reduced serum IL-1β levels and attenuated the infiltration of inflammatory cells in the liver of *E. coli*-infected mice. These data indicated that scutellarin suppressed NLRP3 inflammasome activation in macrophages by augmenting PKA signaling, highlighting its potential therapeutic application for treating NLRP3-related inflammatory diseases.

## Introduction

The NLRP3 (NOD-like receptor family, pyrin domain containing 3; also called cryopyrin and NALP3) is an intracellular sensor that can be activated by a diverse array of factors derived from pathogens, environments and hosts (Guo et al., 2015; Broz and Dixit, 2016; He et al., 2016; Jo et al., 2016; Rathinam and Fitzgerald, 2016). Upon activation, NLRP3 can recruit the adaptor ASC (apoptosis-associated speck-like protein containing a caspase recruitment domain) and procaspase-1 to form a large multiprotein complex named inflammasome, leading to the autocatalytic activation of caspase-1. Subsequently, active caspase-1 in turn converts pro-interleukin (IL)-1β and pro-IL-18 into their mature forms, which are released into the extracellular compartments and act as potent proinflammatory cytokines (He et al., 2016; Jo et al., 2016). Concurrently, active caspase-1 also cleaves gasdermin D to release its active N-terminal fragment which form pores on the plasma membrane thus culminating in pyroptotic cell death and the release of proinflammatory intracellular components into the extracellular milieu (Ding et al., 2016; Liu et al., 2016; Shi et al., 2017). Thus, during pathogenic infections, NLRP3 inflammasomes represent highly proinflammatory platforms that play critical roles in clearing microbial infection and alerting the immune system (de Torre-Minguela et al., 2017).

Macrophages have an important role in mediating the inflammatory responses during infections. Two signals are required to activate the NLRP3 inflammasome in macrophages (He et al., 2016; Jo et al., 2016): The first signal (signal 1) induces the expression of critical inflammasome components including NLRP3 and pro-IL-1β; this signal is mediated by pattern recognition receptors (PRRs) through pathogen-associated molecular patterns (PAMPs). Once such proteins are sufficiently accumulated, the second signal (signal 2) is needed to trigger the assembly of NLRP3 inflammasome. Lipopolysaccharide (LPS) is commonly used PAMPs to confer signal 1, while DAMPs like extracellular ATP (Mariathasan et al., 2006) can act as signal 2 to trigger NLRP3 inflammasome activation in LPS-primed macrophages. ATP can be released from bacteria or host cells during bacterial infections or sterile tissue damages (Piccini et al., 2008; Wegiel et al., 2014), thus being an important triggering signal for NLRP3 inflammasome activation.

Although NLRP3 inflammasomes play critical role in combating against infections, aberrant activation of this inflammasome has been linked to many inflammatory diseases (Guo et al., 2015; de Torre-Minguela et al., 2017). Excessive NLRP3 inflammasome activation results in robust and rapid inflammation due to the release of many proinflammatory cytokines including IL-1β. These massive proinflammatory cytokines in turn enhance pyroptosis leading to multiple organ damage and septic death during bacterial infections (Wree et al., 2014; Kayagaki et al., 2015). In support of this notion, hyperactive NLRP3 due to conditional NLRP3 mutant knock-in culminates in increased hepatocyte pyroptosis and liver injury with decreased mouse survival (Wree et al., 2014). On the other hand, blockade of pyroptosis by caspase-1/-11 or gasdermin D gene deletion renders mice resistant to endotoxin-induced sepsis (Kayagaki et al., 2011, 2015), suggesting NLRP3 and other inflammasome activation play a critical role in sepsis. Excessive activation of NLRP3 inflammasome has also been linked to a variety of inflammatory disorders or metabolic disorders, such as Alzheimer’s disease, atherosclerosis, gout, and obesity (Wen et al., 2012). Intriguingly, NLRP3 depletion dampens the severity of atherosclerosis (Duewell et al., 2010), multiple sclerosis (Shaw et al., 2010), Alzheimer’s disease (Heneka et al., 2013), type 2 diabetes (Vandanmagsar et al., 2011; Wen et al., 2011), and gout (Martinon et al., 2006). Thus, agents targeting NLRP3 activation are promising candidates for the treatment of NLRP3-related diseases.

Scutellarin (4′,5,6-hydroxyl-flavone-7-glucuronide) is a flavonoid purified from *Erigeron breviscapus* (Vant.) Hand.-Mazz. (Gao et al., 2007). This agent has been extensively investigated for its anti-inflammatory and neuroprotective activities (Yuan et al., 2016; Gao et al., 2017). Several studies showed that scutellarin inhibits the expression of inflammatory cytokines, such as tumor necrosis factor (TNF)-α and IL-6, through suppressing the NF-κB pathway in LPS-activated microglia (Wang et al., 2011; Chen et al., 2013) as well as in LPS-induced acute lung injury (Tan et al., 2010). Consistent with these studies, a separate report also showed that oral administration scutellarin significantly attenuated neurological injury in a rat model of ischemia injury, accompanied by reduced expression of TNF-α, IL-1β, and IL-6 (Wang W. et al., 2016). By suppressing the NF-κB and mitogen-activated protein kinase pathways, scutellarin can inhibit RANKL-mediated osteoclastogenesis and titanium particle-induced osteolysis (Zhao et al., 2016). Beyond the NF-κB signaling, the Notch pathway has been identified to be involved in the action of scutellarin on microglia activation (Yuan et al., 2015). Scutellarin has also been shown to attenuate diosbulbin B-induced liver injury by suppressing proinflammatory cytokines (Niu et al., 2015). In clinical practice, scutellarin has been used for the treatment of ischemic cardiovascular and cerebrovascular diseases (Yuan et al., 2016; Gao et al., 2017). The protective effects of scutellarin against hypoxic–ischemic cardiomyocyte and brain injury have been shown to be mediated by its antioxidant capacity (Guo et al., 2011; Wang Z. et al., 2016). As ischemic cerebrovascular diseases involve brain injury and injury-related inflammatory responses (sterile inflammatory responses), it has been proposed that scutellarin may confer neuroprotection by suppressing the activation of microglia (Wang et al., 2011; Yuan et al., 2014, 2015). It is worth noting that during brain injury, endogenous danger signals including ATP are released into the extracellular milieu to activate NLRP3 inflammasomes, thus further exacerbating the injury-related inflammatory responses (Ito et al., 2015). However, it remains unknown whether scutellarin has any influences on the activation of NLRP3 inflammasome in the innate immune cells including macrophages. Its effects on NLRP3-related inflammatory diseases including sepsis also await clarification.

In this study, we found that scutellarin inhibited NLRP3 inflammasome activation and pyroptosis in LPS-primed macrophages upon ATP or nigericin stimulation. ASC speck formation and oligomerization were robustly suppressed by scutellarin. Such scutellarin-mediated suppression of NLRP3 activation and ASC speck formation was abrogated by the protein kinase A (PKA) pathway inhibitors, indicating the involvement of PKA signaling in this process. In further support of this, the Ser/Thr phosphorylation of NLRP3 on PKA-specific sites was markedly increased by scutellarin but was blocked by PKA inhibitor H89. Besides, oral administration of scutellarin significantly attenuated systemic inflammation and improved the survival of mice with bacterial sepsis, suggesting that it could inhibit NLRP3 activation *in vivo*. Our data reveals that scutellarin is able to suppress NLRP3 inflammasome activation thus exhibiting therapeutic effects against NLRP3-related inflammatory diseases.

## Materials and Methods

### Reagents

Scutellarin (≥98% purity) (110842) was obtained from Guangdong Institute for Drug Control (Guangzhou, China). Dulbecco’s Modified Eagle’s Medium (DMEM) medium with high glucose, Opti-MEM, fetal bovine serum (FBS), streptomycin and penicillin were products of ThermoFisher/Gibco (Carlsbad, CA, United States). MDL12330A (M182), adenosine triphosphate (ATP) (A6419), lipopolysaccharide (LPS) (*Escherichia coli* O111:B4) (L4391), dimethyl sulfoxide (DMSO) (D8418), propidium iodide (P4170), Hoechst 33342 (B2261), Tween 80 (P8074) and disuccinimidyl suberate (S1885) were purchased from Sigma–Aldrich (St. Louis, MO, United States). H89 (S1643), cell lysis buffer for Western and IP (P0013) and phenylmethanesulfonyl fluoride (PMSF) (ST505) were bought from Beyotime Biotechnology (Haimen, China). Nigericin (#tlrl-nig) was purchased from InvivoGen (San Diego, CA, United States). The antibody against caspase-1p10 (sc-514) was purchased from Santa Cruz (Dallas, TX, United States). The antibody against NLRP3 (Cryo-2) (AG-20B-0014) was purchased from Adipogen AG (Liestal, Switzerland). Specific antibodies against IL-1β (#12242), HMGB1 (#3935), ASC (#67824), p-(Ser/Thr) PKA substrate (#9621), and β-tubulin (#2128) were bought from Cell Signaling Technology (Danvers, MA, United States). The horse-radish peroxidase (HRP)-conjugated horse anti-mouse IgG (#7076), HRP-conjugated goat-anti-rabbit IgG (#7074) and Protein G Agarose beads (#37478) were also from Cell Signaling Technology. CF568 goat-anti-rabbit IgG (H + L), highly cross-adsorbed (#20103) and CF488A-conjugated goat-anti-mouse IgG (#20018), highly cross-adsorbed were obtained from Biotium (Hayward, CA, United States). Scutellarin was dissolved in DMSO at 100 mM, and stored at -20°C.

### Experimental Mice

Female C57BL/6 mice (6–8 weeks of age) were purchased from the Experimental Animal Center of Southern Medical University (Guangzhou, China). Mice were acclimatized for 1 week before experiments. All animal experiments were performed in accordance with the guidelines for the care and use of animals approved by the Committee on the Ethics of Animal Experiments of Jinan University (approved No. JNU20160315).

### Macrophage Culture

Bone marrow-derived macrophages (BMDMs) were differentiated as described previously (Kayagaki et al., 2011; Li et al., 2017). In brief, C57BL/6 mice were sacrificed and bone marrow cells were differentiated in culture medium (DMEM supplemented with 10% FBS, 100 U/ml penicillin, 100 μg/ml streptomycin and 20% M-CSF-conditioned medium) for 6 days at 37°C in a humidified incubator of 5% CO_2_. BMDMs were cultured in 24-well plates at 1.5 × 10^5^ cells/well (0.5 ml) or in 6-well plates at 1.2 × 10^6^ cells/well (2 ml) or in 10-cm petri dish at 7.5 × 10^6^ cells/dish (10 ml) with complete DMEM medium (DMEM supplemented with 10% FBS, 100 U/ml penicillin, and 100 μg/ml streptomycin) at 37°C overnight.

### Cell Death Assay

Cell death was measured by propidium iodide (PI) incorporation as described previously (Py et al., 2014; Li et al., 2017). Briefly, BMDMs were primed in Opti-MEM with 500 ng/ml LPS for 4 h. Then the cells were treated with different doses of scutellarin for 1 h followed by ATP (3 mM) or nigericin (10 μM) for indicated time periods. The nuclei were revealed by Hoechst 33342 (5 μg/ml) staining and dead cells revealed by PI (2 μg/ml) staining at room temperature for 10 min. The cells were observed immediately by live imaging using Zeiss Axio Observer D1 microscope equipped with a Zeiss LD PlanNeofluar 20×/0.4 Korr M27 objective lens (Carl Zeiss MicroImaging GmbH, Göttingen, Germany). Fluorescence images were captured with a Zeiss AxioCam MR R3 cooled CCD camera controlled with ZEN software (Carl Zeiss).

### Precipitation of Soluble Proteins

Soluble protein in culture supernatants was precipitated as previously described (Kayagaki et al., 2011; Li et al., 2017). Briefly, soluble proteins in culture supernatants (equal volume for each sample) were precipitated with 7.2% trichloroacetic acid plus 0.15% sodium deoxycholate. The precipitated proteins were re-dissolved in 2× sodium dodecylsulfate-polyacrylamide gel electrophoresis (SDS-PAGE) sample loading buffer and subjected to western blot analysis for released mature IL-1β, caspase-1p10 and HMGB1.

### Immunoblotting

Immunoblotting was performed essentially as previously described (Lin et al., 2016). In brief, equal amounts of proteins were separated by SDS-PAGE followed by electrotransfer to polyvinylidene difluoride (PVDF) membranes (#03010040001; Roche Diagnostics GmbH, Mannheim, Germany). Membranes were blocked and incubated with indicated primary antibodies at 4°C overnight, followed by incubation with appropriate HRP-conjugated secondary antibody. Bands were revealed by a BeyoECL Plus kit (P0018; Beyotime, Haimen China) and recorded on X-ray films (Carestream, Xiamen, China). The densitometry of each band was quantified by FluorChem 8000 (Alpha Innotech; San Leandro, CA, United States).

### Soluble IL-1β Detection

Soluble cytokine IL-1β in culture supernatants of BMDMs was detected by Cytometric Bead Array (CBA) Mouse IL-1β Flex Set (BD Biosciences, San Jose, CA, United States) according to the manufacturer’s instructions. Data were acquired on a flow cytometer (FACSCalibur; Becton Dickinson) equipped with CELLQuest Pro software (Becton Dickinson).

### Immunofluorescence Microscopy

Immunofluorescence analysis was performed as previously described (Zhao et al., 2015; Zha et al., 2016). Briefly, BMDMs were seed in glass-bottomed dishes (4 × 10^5^ cells/dish) and cultured in complete DMEM medium at 37°C overnight. Cells were primed in Opti-MEM with 500 ng/ml LPS for 4 h, and then treated with different doses of scutellarin for 1 h followed by treatment with 3 mM ATP for 30 min or 10 μM nigericin for 1 h in Opti-MEM. After fixation, permeabilization and blocking, the cells were incubated with anti-ASC antibody (1:300) and anti-NLRP3 antibody (1:300) overnight at 4°C, followed by staining with CF568-conjugated goat-anti-rabbit IgG and CF488A-conjugated goat-anti-mouse IgG. Nuclei were revealed by Hoechst 33342 and ASC speck formation was observed under a Zeiss Axio Observer D1 microscope with a Zeiss LD Plan-Neofluar 40×/0.6 Korr M27 objective (Carl Zeiss MicroImaging GmbH, Göttingen, Germany). Fluorescence images were captured by a Zeiss AxioCam MR R3 cooled CCD camera controlled with ZEN software (Carl Zeiss).

### ASC Oligomerization

The cross-linking of ASC oligomers was performed as described previously (He et al., 2014; Coll et al., 2015). Briefly, BMDMs were seeded in 6-well plates at 1.0 × 10^6^ cells/well. After appropriate treatments, cells were lysed with cold PBS containing 0.5% Triton X-100, and the cell lysates were centrifuged at 6000 × *g* for 15 min at 4°C. The pellets were washed twice with PBS and then re-suspended in 200 μl PBS. Freshly prepared disuccinimidyl suberate (2 mM) was added to the re-suspended pellets and the suspension was incubated at room temperature for 30 min with rotation. The cross-linked pellets were collected by centrifugation at 6000 × *g* for 15 min at 4°C and re-dissolved in 25 μl of 1× SDS-PAGE sample loading buffer. Samples were boiled for 5 min and subjected to western blot analysis.

### Immunoprecipitation

After appropriate treatments, cells were rinsed once with ice-cold PBS and lyzed with 0.5 ml ice-cold cell lysis buffer for Western blot and IP (containing 1 mM PMSF). Cell lysates were centrifuged at 13,000 × *g* for 10 min at 4°C and pre-cleared with a 10% volume of Protein G agarose beads for 30 min at 4°C with gentle agitation. The pre-cleared cell lysates were then incubated with anti-NLRP3 antibody (1:200) overnight at 4°C with gentle shaking. Antibody-NLRP3 complexes were collected with a 10% volume of Protein G agarose beads for 2 h at 4°C with gentle shaking. The beads were washed five times with cell lysis buffer, boiled for 5 min in 3× SDS-PAGE sample loading buffer, and resolved by Western blot analysis.

### Bacterial Infection

The murine model of bacterial sepsis was established according to the protocol described previously (Wegiel et al., 2014; Zha et al., 2016). In brief, thirty C57BL/6 mice were acclimated for 1 week, randomly divided into three groups (10 mice/group), and intragastrically administered once with scutellarin solution (100 mg/kg or 200 mg/kg body weight) or vehicle (2% Tween-80 in PBS), which has referred to previous studies (Li et al., 2011; Yuan et al., 2014; Wang W. et al., 2016). Three hour later, viable *E. coli* (DH5α) cells (2.5 × 10^9^ colony-forming units (CFU)/mouse, in 0.5 ml of PBS) were injected into the peritoneal cavity of each mouse. One hour after bacterial infection, mice were intragastrically administered once again with scutellarin solution or vehicle, respectively. Mouse survival was monitored every 6 h for 5 consecutive days.

In another experiment, 54 mice (6 mice/group) were treated with scutellarin (100 or 200 mg/kg body weight) or vehicle once by oral gavage, respectively. Three hour later, viable *E. coli* cells (2.0 × 10^9^ CFU/mouse, in 0.5 ml of PBS) were injected into the peritoneal cavity of all mice. One hour later, mice were gavaged once again with scutellarin or vehicle, respectively. The mice were sacrificed 4 and 8 h after bacterial infection, respectively. Their sera were collected, and serum IL-1β levels were measured by CBA mouse IL-1β Flex Set. The bacterial loads were measured as described previously (Pan et al., 2015). The liver and intestine were isolated and fixed in 4% neutral formaldehyde. Paraffin slices of the tissues were stained with hematoxylin and eosin. Images were captured under the Zeiss Axio Observer D1 microscope armed with a color CCD (ZEISS).

### Statistical Analysis

Experiments were performed three times independently, with one representative experiment shown. Data were expressed as mean ± standard deviation (SD). Statistical analysis was performed using GraphPad Prism5.0 (GraphPad Software Inc, San Diego, CA, United States). One-way analysis of variance (ANOVA) followed by Tukey *post hoc* test and unpaired Student’s *t*-test were used to analyze the statistical significance among multiple groups and between two groups, respectively. Kaplan–Meier survival curves were used for analysis of mouse survival, and the statistical difference between 2 groups was determined using the log-rank (Mantel-Cox) test. *P*-value < 0.05 was considered statistically significant.

## Results

### Scutellarin Suppressed ATP or Nigericin-Induced Caspase-1 Activation and IL-1β Secretion in Macrophages

**FIGURE 1:**
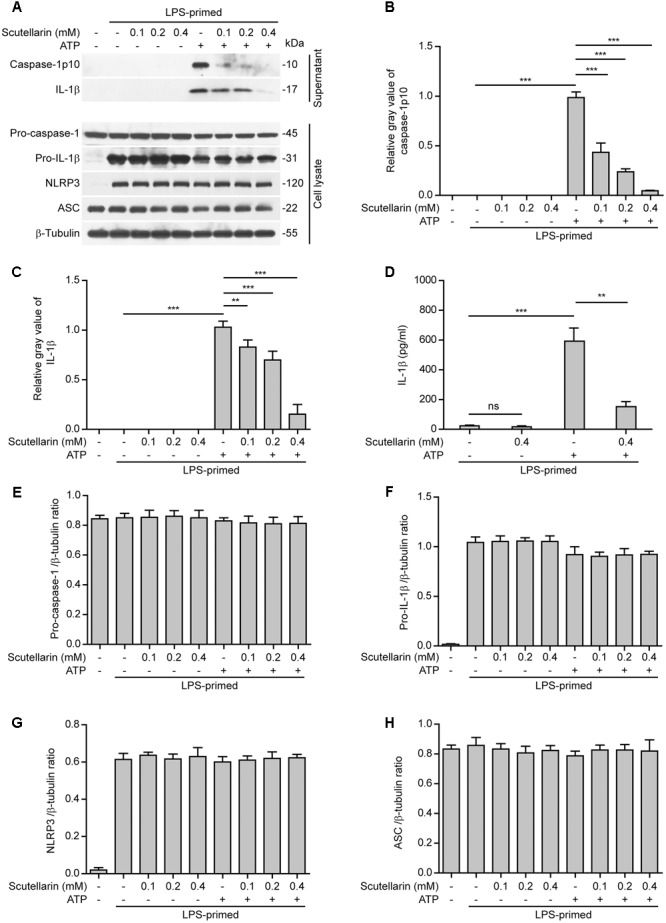
Scutellarin inhibited ATP-induced caspase-1 activation and mature IL-1β secretion in macrophages. Bone marrow-derived macrophages (BMDMs) were first primed with LPS (500 ng/ml) for 4 h, and then pre-treated with indicated concentration of scutellarin for 1 h, followed by stimulation with ATP (3 mM) for 30 min without LPS. **(A)** Western blot analysis of caspase-1 and IL-1β in the culture supernatants and indicated proteins in cell lysates. β-Tubulin was used as a loading control for cell lysates. See Supplementary Material for source blots. **(B,C)** Histograms showing the relative gray values of capase-1p10 **(B)** or mature IL-1β **(C)** in **(A)**. The gray value of capase-1p10 or IL-1β bands in ATP group was set to 1.0 with those of the other groups being calculated relatively to the ATP group. **(D)** Cells were treated as in **(A)**. Cytometric bead array (CBA) was used to assay IL-1β levels in the culture supernatants. Data are shown as mean ± SD (*n* = 3). **(E–H)** Histograms showing the ratios of gray values of indicated proteins relative to β-tubulin in **(A)** (*n* = 3). ^∗∗^*P <* 0.01; ^∗∗∗^*P <* 0.001; ns, not significant.

By using bone marrow-derived macrophages (BMDMs) primed with LPS, we initially sought to explore the influence of scutellarin on NLRP3 inflammasome activation upon canonical NLRP3 activator stimulation. Western blot analysis showed that ATP treatment of LPS-primed macrophages induced a rapid release of active caspase-1p10 (10 kDa) and mature IL-1β (17 kDa) into culture supernatants, indicative of NLRP3 inflammasome activation. Notably, scutellarin pre-treatment dose-dependently reduced ATP-induced release of active caspase-1p10 and mature IL-1β from macrophages, whereas scutellarin *per se* did not induce the release of these proteins (**Figures 1A–C**). Consistent with previous studies (Kayagaki et al., 2013), the pro-IL-1β and NLRP3 proteins were highly expressed after LPS priming, whereas pro-caspase-1 and ASC were constitutively expressed regardless of LPS priming in BMDMs (**Figure 1A**). We also detected the mature IL-1β levels in the culture supernatants by using a bead-based immunoassay (CBA), and found that scutellarin markedly reduced IL-1β release upon ATP stimulation (**Figure 1D**), thus confirming the result of western blot analysis of IL-1β release (**Figure 1A**). Besides, scutellarin had minimal effects on the expression of pro-caspase-1, pro-IL-1β, NLRP3, and ASC in the cell lysates (**Figures 1A,E–H**). These results indicated that scutellarin inhibited NLRP3 inflammasome activation in macrophages upon ATP treatment.

**FIGURE 2:**
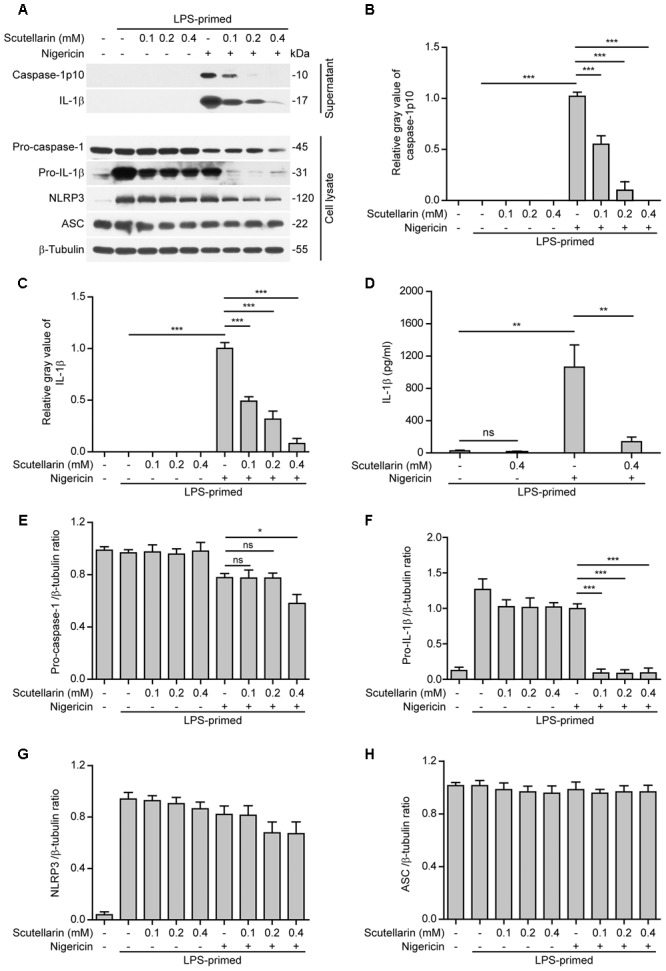
Scutellarin inhibited nigericin-induced caspase-1 activation and mature IL-1β secretion. Bone marrow-derived macrophages (BMDMs) were first primed with LPS (500 ng/ml) for 4 h, and then pre-treated with indicated doses of scutellarin for 1 h, followed by incubation with nigericin (10 μM) for 1 h in the absence of LPS. **(A)** Western blot analysis of indicated proteins in culture supernatants and cell lysates. β-Tubulin was recruited as a loading control for cell lysates. See Supplementary Material for source blots. **(B,C)** Histograms showing the relative gray values of capase-1p10 **(B)** or mature IL-1β **(C)** in **(A)** (*n* = 3). The gray value of capase-1p10 or IL-1β bands in nigericin group was set to 1.0 and those of the other groups were relative to the ATP group. **(D)** Cells were treated as in **(A)**. Cytometric bead array (CBA) was used to measure IL-1β levels in the culture supernatants. Data are shown as mean ± SD (*n* = 3). **(E–H)** Histograms showing the ratios of gray values of indicated proteins relative to β-tubulin in **(A)** (*n* = 3). ^∗^*P <* 0.05; ^∗∗^*P <* 0.01; ^∗∗∗^*P <* 0.001; ns, not significant.

Apart from extracellular ATP, nigericin is another commonly used activator for the NLRP3 inflammasome (He et al., 2016). Different from ATP that acts on the plasma membrane P2X7 purinergic receptor to induce K^+^ efflux thus triggering NLRP3 activation, nigericin is a potassium ionophore thus bypassing the P2X7 receptor to trigger NLRP3 inflammasome activation (Mariathasan et al., 2006). Similar to those results obtained from ATP-treated macrophages (**Figure 1**), scutellarin dose-dependently reduced caspase-1p10 and mature IL-1β secretion from LPS-primed macrophages upon nigericin stimulation (**Figures 2A–D**), suggesting that it suppressed nigericin-induced NLRP3 activation. Different from ATP stimulation, under nigericin stimulation, scutellarin treatment drastically reduced pro-IL-1β expression while also moderately decreasing the expression of pro-caspase-1 but not NLRP3 and ASC in the cell lysates (**Figures 2A,E–H**); the reason underlying this difference is unclear and needs further investigation. Together, these results indicated that scutellarin inhibited both ATP- and nigericin-induced NLRP3 inflammasome activation in murine macrophages.

### Scutellarin Inhibited Pyroptosis in Macrophages upon NLRP3 Activation

**FIGURE 3:**
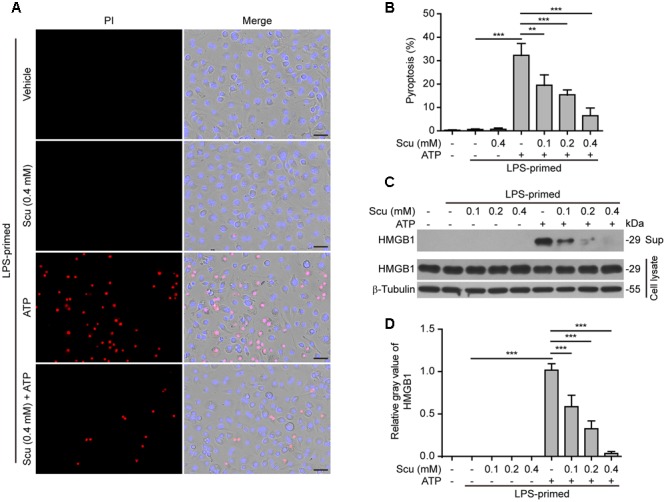
Scutellarin inhibited ATP-induced pyroptosis in macrophages. Bone marrow-derived macrophages (BMDMs) were treated as in **Figure 1A**. **(A)** Cell death was measured by staining with propidium iodide (PI) (red, staining dead cells) and Hoechst 33342 (blue, staining all cells) for 10 min. All images were captured by fluorescence microscopy, and the merged images show PI and Hoechst 33342 fluorescence with bright-field images. One set of representative images of three independent experiments is shown. Scale bars, 50 μm. **(B)** PI-positive cells in 5 randomly chosen fields each containing ∼100 cells were quantified. The percentage of pyroptosis is defined as the ratio of PI-positive relative to all (revealed by Hoechst) cells. Data are shown as mean ± SD (*n* = 5). **(C)** Cells were treated as in **(A)**. Western blotting was used to assess the expression levels of indicated proteins in the cell lysates and culture supernatants (Sup), respectively. β-Tubulin was adopted as a loading control for cell lysates. See Supplementary Material for source blots. **(D)** Histograms showing the relative gray values of HMGB1 in **(C)**. The gray value of HMGB1 band in ATP group was set to 1.0 and those of the other groups were relative to the ATP group. Data are shown as mean ± SD (*n* = 3). ^∗∗^*P <* 0.01; *^∗∗∗^P* < 0.001; Scu, scutellarin.

**FIGURE 4:**
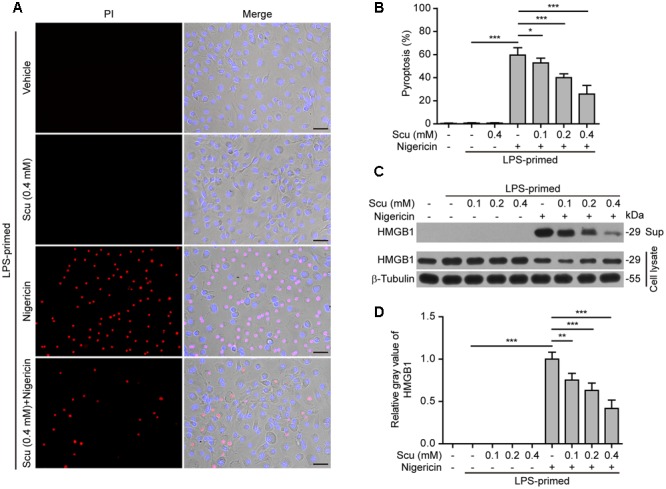
Scutellarin inhibited nigericin-induced pyroptosis in macrophages. Bone marrow-derived macrophages (BMDMs) were treated as in **Figure 2A**. **(A)** Cell death was measured by staining with propidium iodide (PI) (red, staining dead cells) and Hoechst 33342 (blue, staining all cells) for 10 min. Fluorescent images were captured by fluorescence microscopy, and merged with bright-field images. One set of representative images of three independent experiments is shown. Scale bars, 50 μm. **(B)** PI-positive cells in 5 randomly chosen fields each containing ∼100 cells were quantified. The percentage of pyroptosis is defined as the ratio of PI-positive relative to all (revealed by Hoechst) cells. Data are shown as mean ± SD (*n* = 5). **(C)** Cells were treated as in **(A)**. Western blotting was used to assess the expression levels of indicated proteins in the cell lysates and culture supernatants (Sup), respectively. β-Tubulin was used as a loading control for cell lysates. See Supplementary Material for source blots. **(D)** Histograms showing the relative gray values of HMGB1 in **(C)**. The gray value of HMGB1 band in nigericin group was set to 1.0 with those of the other groups being calculated relative to the nigericin group. Data are shown as mean ± SD (*n* = 3). ^∗^*P <* 0.05; ^∗∗^*P <* 0.01; *^∗∗∗^P* < 0.001; Scu, scutellarin.

As NLRP3 inflammasome activation results in caspase-1 activation leading to pyroptosis and release of danger signals including HMGB1, we next explored whether scutellarin had any effect on ATP- or nigericin-induced pyroptosis in LPS-primed BMDMs. The pyroptotic cell death was revealed by propidium iodide (PI) staining. As shown in **Figures 3A,B**, ATP treatment induced pyroptosis in ∼35% of the cells. Notably, when compared with ATP alone group, scutellarin treatment before ATP stimulation markedly reduced pyroptosis rates in a dose-dependent manner. To further corroborate this, scutellarin also dose-dependently reduced the release of HMGB1 from the macrophages into the culture supernatants (**Figures 3C,D**). Without ATP triggering, however, scutellarin alone induced neither pyroptosis nor HMGB1 release in these cells. Similarly, scutellarin dose-dependently reduced nigericin-induced pyroptosis in LPS-primed macrophages (**Figures 4A,B**), and HMGB1 release into the culture supernatants (**Figures 4C,D**). Together these results showed that scutellarin treatment inhibited NLRP3 inflammasome activation and pyroptosis in LPS-primed murine macrophages upon ATP or nigericin stimulation.

### NLRP3-Mediated Formation of ASC Specks in Macrophages Was Blocked by Scutellarin

**FIGURE 5:**
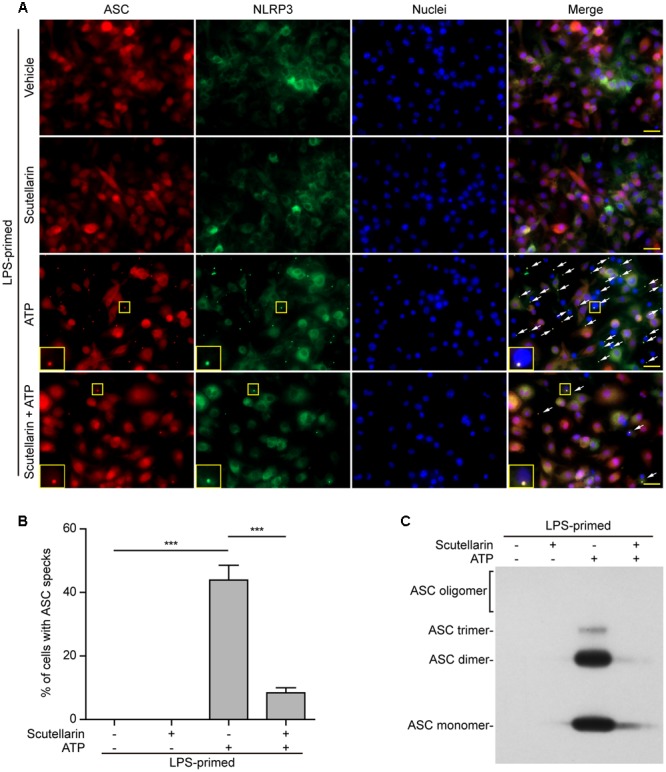
Scutellarin blocked ATP-induced ASC speck formation and oligomerization in macrophages. Bone marrow-derived macrophages (BMDMs) were first primed with LPS (500 ng/ml) for 4 h, and then the cells pre-treated with scutellarin (400 μM) for 1 h, followed by incubation with ATP (3 mM) for 30 min without LPS. **(A)** Representative immunofluorescence images showing ASC (red) and NLRP3 (green) subcellular distribution. Nuclei (blue) were revealed by Hoechst 33342. The merged images are presented to show the co-localization of ASC with NLRP3. White arrows indicate ASC specks and the enlarged inset showing cells with an ASC speck. Scale bars, 20 μm. **(B)** Percentages of cells with an ASC speck relative to total cells from 5 random fields each containing ∼50 cells. Data are shown as mean ± SD (*n* = 3). *^∗∗∗^P <* 0.001. **(C)** Western blot analysis of disuccinimidyl suberate-cross-linked pellets with anti-ASC antibody, showing ASC oligomerization in each sample.

**FIGURE 6:**
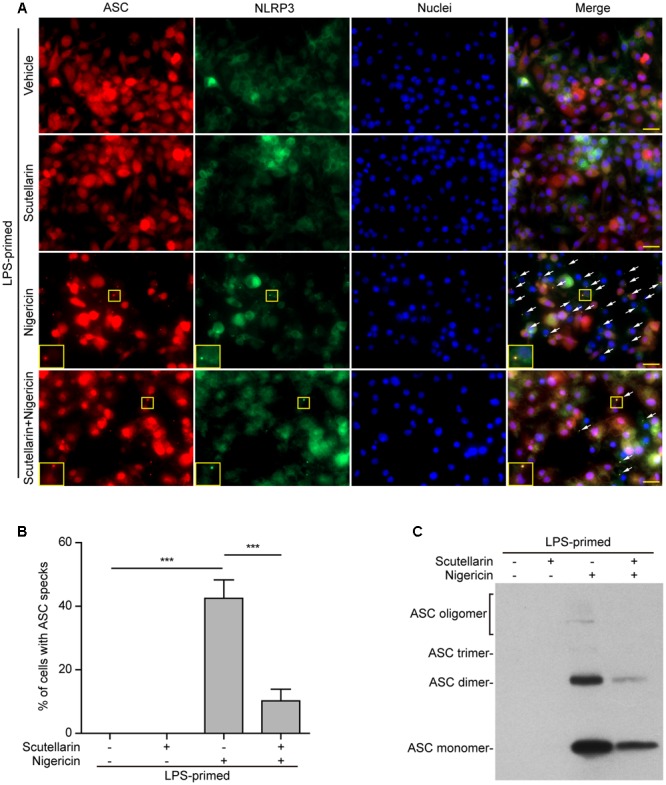
Scutellarin blocked nigericin-induced ASC speck formation and oligomerization in macrophages. Bone marrow-derived macrophages (BMDMs) were first primed with LPS (500 ng/ml) for 4 h, and then pre-treated with scutellarin (400 μM) for 1 h, followed by incubation with nigericin (10 μM) for 1 h without LPS. **(A)** Representative immunofluorescence images showing ASC (red) and NLRP3 (green) subcellular distribution. Nuclei (blue) were revealed by Hoechst 33342. The merged images are presented to show the co-localization of ASC with NLRP3. White arrows indicate ASC specks and the insets show cells with an ASC speck. Scale bars, 20 μm. **(B)** Quantification of ASC speck formation by the number of cells with ASC specks relative to the total number of cells from 5 random fields each containing ∼50 cells. Data are shown as mean ± SD (*n* = 3). *^∗∗∗^P <* 0.001. **(C)** Western blotting of disuccinimidyl suberate-cross-linked pellets with anti-ASC antibody, showing ASC oligomerization in each sample.

Upon treatment with NLRP3 activators, the adaptor ASC is recruited by NLRP3 to form one large speck in each macrophage, which can be revealed by immunofluorescence microscopy. The results showed that ASC was diffusely distributed in LPS or LPS with scutellarin-treated cells. Upon ATP or nigericin treatment, ASC specks were formed in ∼45% or ∼40% of the cells, whereas scutellarin pre-treatment before ATP or nigericin stimulation markedly reduced the percentages of cells containing ASC specks to 10% or 3%, respectively (**Figures 5A,B, 6A,B**). Consistent with previous reports (Wang et al., 2013; Granata et al., 2015), ASC specks were highly co-localized with NLRP3 in the cytoplasm, indicative of the recruitment of ASC by NLRP3 (**Figures 5A, 6A**). These results indicated that scutellarin inhibited NLRP3 activation by blocking ASC speck formation.

Apart from assaying ASC speck formation, we used chemical cross-linking to detect ASC oligomerization, which also reflects the activation of NLRP3 inflammasome (He et al., 2014; Coll et al., 2015). Different ASC multimers (dimers, trimers and higher oligomers) together with monomers were detected in ATP- or nigericin-treated macrophages, but were not detectable in both vehicle- and scutellarin-treated cells without ATP or nigericin. Similar to ASC speck assay, ATP- or nigericin-induced formation of ASC multimers was markedly attenuated by scutellarin pre-treatment (**Figures 5C, 6C**). Therefore, these data further confirmed that scutellarin inhibited NLRP3 inflammasome activation by blocking ASC recruitment to form ASC specks in macrophages.

### Blocking PKA Signaling Reversed Scutellarin-Mediated Suppression of ASC Specks

**FIGURE 7:**
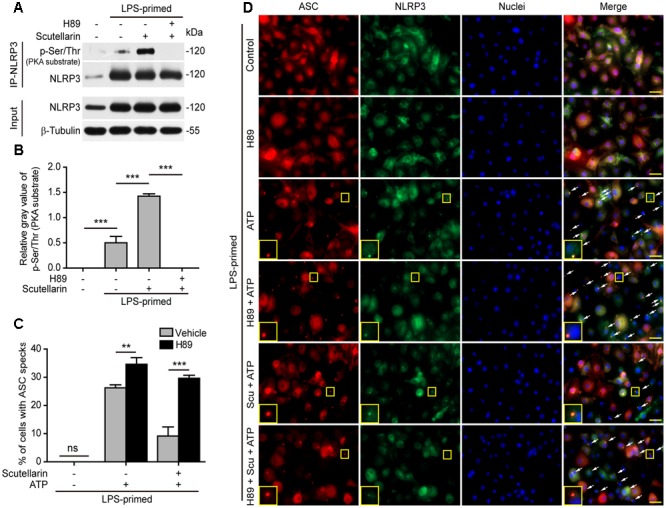
Scutellarin inhibited NLRP3 inflammasome activation via PKA signaling. **(A)** LPS-primed BMDMs were incubated with the selective PKA inhibitor H89 (20 μM) for 30 min, and then treated with scutellarin (400 μM) for 1 h. NLRP3 was immunoprecipitated from the cell lysates and analyzed for its Ser/Thr phosphorylation on PKA-specific sites (p-Ser/Thr). See Supplementary Material for source blots. **(B)** The gray values of NLRP3 phosphorylation on PKA-specific sites (p-Ser/Thr) relative to total NLRP3 in **(A)**. **(C)** BMDMs were treated as in **Figure 5**. Immunofluorescence images were obtained to show ASC (red) and NLRP3 (green) subcellular distribution. Nuclei (blue) were revealed by Hoechst 33342. White arrows indicate ASC specks and the insets show enlarged cells with an ASC speck. Scale bars, 20 μm. **(D)** Percentages of cell containing an ASC speck relative to the total cells from 5 random fields each containing ∼50 cells. *^∗∗^P* < 0.01; *^∗∗∗^P <* 0.001; ns, not significant; Scu, scutellarin.

The above-mentioned data indicated that scutellarin suppressed NLRP3 inflammasome activation by blocking ASC speck formation. As PKA signaling has been recently demonstrated to phosphorylate and suppress NLRP3 activation (Yan et al., 2015; Guo et al., 2016; Mortimer et al., 2016), we next explored whether PKA signaling was involved in scutellarin-mediated suppression of ASC speck formation upon NLRP3 inflammasome activation. We thus sought to determine whether scutellarin could enhance phosphorylation of NLRP3 on PKA-specific sites. A specific antibody against p-Ser/Thr PKA substrate was used to analyze NLRP3 phosphorylation. The results showed that scutellarin treatment significantly enhanced NLRP3 phosphorylation on PKA-specific sites in LPS-primed macrophages. Further, scutellarin-mediated augmentation of NLRP3 phosphorylation was completely abrogated by PKA inhibitor H89 (**Figures 7A,B**).

Next, we detected ASC speck formation in the presence or absence of PKA inhibitor H89 by immunofluorescence microscopy. Notably, blocking PKA signaling by H89 not only significantly enhanced ATP-induced ASC speck formation, but also completely abrogated scutellarin-mediated suppression of ATP-induced ASC speck formation in macrophages (**Figures 7C,D**). H89 alone did not induce ASC speck formation. These data indicated that scutellarin inhibited ASC speck formation upon NLRP3 inflammasome activation by regulating PKA signaling. Together, these results indicated that scutellarin-induced inhibition of ASC speck formation upon NLRP3 inflammasome activation was mediated by PKA signaling.

### Blockade of PKA Signaling Abrogated Scutellarin-Mediated Suppression of NLRP3 Inflammasome Activation and Pyroptosis

**FIGURE 8:**
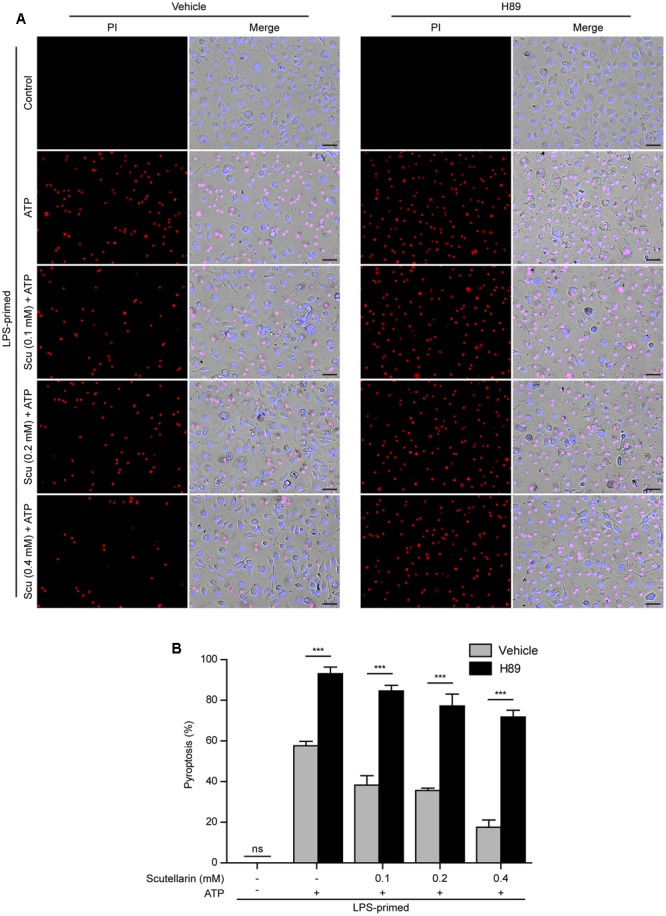
Scutellarin-mediated suppression of NLRP3 inflammasome activation was abrogated by PKA inhibitor. LPS-primed bone marrow-derived macrophages (BMDMs) were pre-treated with the selective PKA inhibitor H89 (20 μM) for 30 min, and then incubation with indicated doses of scutellarin for 1 h, followed by stimulation with ATP (3 mM) for 30 min. **(A)** Cell death was assayed by propidium iodide (PI) (red) and Hoechst 33342 (blue) co-staining for 10 min. The images were captured by fluorescence microscopy. One set of representative images of three independent experiments is shown. Scale bars, 50 μm. **(B)** PI-positive cells in 5 randomly chosen fields each containing ∼100 cells were quantified. The percentage of pyroptosis is defined as the ratio of PI-positive relative to all (revealed by Hoechst) cells. Data are shown as mean ± SD (*n* = 5). *^∗∗∗^P <* 0.001; ns, not significant; Scu, scutellarin.

We next investigated the role of PKA signaling induced by scutellarin on NLRP3 activation and pyroptosis. BMDMs were primed with LPS, pre-treated with the selective PKA inhibitor H89, and then co-incubated with scutellarin followed by ATP treatment to induce NLRP3 inflammasome activation and pyroptosis. The results showed that ATP-induced pyroptosis was markedly enhanced by H89 treatment. Of note, scutellarin-mediated suppression of ATP-induced pyroptosis was completely abrogated by H89 treatment (**Figures 8A,B**). These results suggested that scutellarin-induced inhibition of NLRP3 activation was dependent on PKA signaling.

**FIGURE 9:**
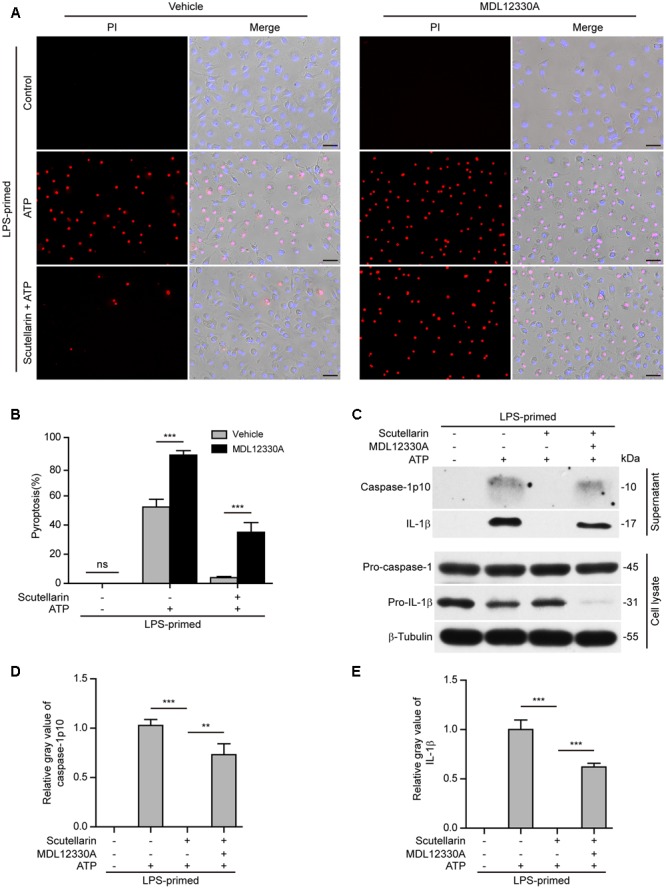
Scutellarin-mediated suppression of NLRP3 inflammasome activation was blocked by adenyl cyclase inhibitor. Bone marrow-derived macrophages (BMDMs) were primed with LPS (500 ng/ml) for 4 h, and pre-treated with scutellarin (400 μM) for 30 min before incubation with inhibitor MDL12330A (10 μM) for 30 min, followed by co-treatment with ATP (3 mM) for 30 min. **(A)** Cell death was measured by staining with propidium iodide (PI) (red, staining dead cells) and Hoechst 33342 (blue, staining all cells) for 10 min. Fluorescent images were captured by fluorescent microscopy, and merged with bright-field images. One set of representative images of three independent experiments is shown. Scale bars, 50 μm. **(B)** PI-positive cells in 5 randomly chosen fields each containing ∼100 cells were quantified. The percentage of pyroptosis is defined as the ratio of PI-positive relative to all (revealed by Hoechst) cells. Data are shown as mean ± SD (*n* = 5). **(C)** Western blot analysis was used to detect the expression and secretion of caspase-1 and IL-1β in the cell lysates and culture supernatants, respectively. β-Tubulin was used as a loading control for cell lysates. See Supplementary Material for source blots. **(D**,**E)** Histograms showing the relative gray values of capase-1p10 **(D)** or IL-1β **(E)** in **(C)**. The gray value of capase-1p10 or IL-1β bands in ATP group was set to 1.0 and those of the other groups were calculated relative to the ATP group. Data are shown as mean ± SD (*n* = 3). *^∗∗^P <* 0.01; *^∗∗∗^P <* 0.001; ns, not significant; Scu, scutellarin.

Further, we sought to explore whether adenyl cyclase (the upstream component of PKA signaling) also blocked scutellarin’s inhibitory action on NLRP3 inflammasome activation. The specific adenyl cyclase inhibitor MDL12330A was used to block cAMP production. Similar to H89 treatment, MDL12330A not only increased ATP-induced pyroptosis but also significantly reversed scutellarin-mediated suppression of ATP-induced pyroptosis (**Figures 9A,B**). Consistent with this, scutellarin-mediated suppression of caspase-1p10 and mature IL-1β (17 kDa) release into the culture supernatants of BMDMs were also markedly reversed by MDL12330A treatment (**Figures 9C–E**). Together, these results indicated that scutellarin inhibited NLRP3 inflammasome activation and pyroptosis by augmenting PKA signaling in murine macrophages.

### Scutellarin Administration Protected Mice against Bacterial Sepsis

**FIGURE 10:**
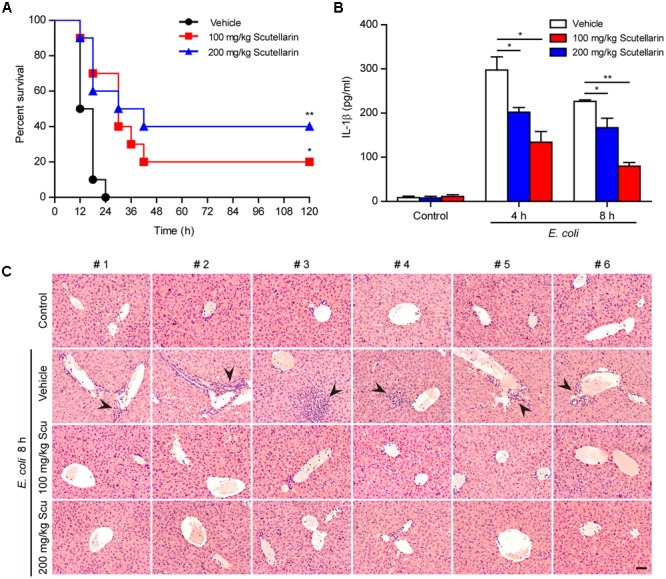
Scutellarin administration improved the survival of mice with bacterial sepsis. C57BL/6 mice were administered once intragastrically (i.g.) with scutellarin (100 or 200 mg/kg body weight) or vehicle (2% Tween 80 in PBS) 3 h prior to bacterial infection intraperitoneally (i.p.) with viable *Escherichia coli* (2.5 × 10^9^ CFU/mouse). Mice were administered (i.g.) with scutellarin or vehicle once again at 1 h after the bacterial injection. Mouse survival was monitored every 6 h for 5 consecutive days. Kaplan–Meier survival curves were used to analyze the data (10 mice per group). The significance was evaluated by the log-rank (Mantel-Cox) test. **(B)** Mice were treated as in **(A)** except that mice were injected (i.p.) with viable *E. coli* (2.0 × 10^9^ CFU/mouse). The serum levels of IL-1β at 4 and 8 h post bacterial infection were measured by CBA assay (6 mice per group). Data are shown as mean ± SD (*n* = 6). *^∗^P <* 0.05; *^∗∗^P <* 0.01. **(C)** Hematoxylin and eosin staining of the liver of mice infected with live *E. coli* for 8 h. Representative images from each mouse were presented and arrowheads indicated infiltrated inflammatory cells. The numbers on the top indicate mouse number. Scale bar, 50 μm; Scu, scutellarin.

As ATP-induced NLRP3 activation and pyroptosis plays a critical role in sepsis of bacterial infection (Wegiel et al., 2014), we finally explored whether scutellarin could attenuate bacterial sepsis in a mouse model of intraperitoneal infection. As shown in **Figure 10A**, mice infected with viable *E. coli* (2.5 × 10^9^ CFU/mouse) died within 24 h, whereas oral administration of scutellarin significantly prolonged mouse survival of bacterial sepsis. When administered with low-dose (100 mg/kg) and high-dose (200 mg/kg) scutellarin, 20 and 40% of mice had survived the period of observation (120 h), respectively (**Figure 10A**). In addition, scutellarin treatment significantly reduced serum IL-1β levels and attenuated infiltration of inflammatory cells in the liver of mice infected with *E. coli* in comparison to vehicle (**Figures 10B,C**), indicating that scutellarin inhibited systemic inflammation in the mice. But there was no obvious lesion in the jejunum and colon tissues with or without scutellarin administration (Supplementary Figure S1). Scutellarin had no influences on IL-1β levels in uninfected (control) mice (**Figure 10B**). The bacterial loads were also unchanged by scutellarin administration (Supplementary Figure S2). As IL-1β secretion *in vivo* upon Gram-negative bacterial infection has been shown to be NLRP3 dependent (He et al., 2013), these results suggested that scutellarin protected mice against bacterial sepsis probably by suppressing NLRP3 inflammasome activation upon microbial infection.

## Discussion

Scutellarin has been shown to have potent anti-inflammatory activities (Tan et al., 2010; Wang et al., 2011; Chen et al., 2013; Yuan et al., 2014; Niu et al., 2015; Wang W. et al., 2016; Zhao et al., 2016). But it was unclear whether this agent has any potential effects on NLRP3 inflammasome activation, which has been implicated in many inflammatory conditions ranging from metabolic disorders to bacterial sepsis. In the current study, we found that scutellarin robustly inhibited ATP- or nigericin-induced NLRP3 inflammasome activation and pyroptosis in murine macrophages, thus unraveling a previously unappreciated action mechanism for scutellarin in preventing inflammatory responses in tissue injury and bacterial infections.

NLRP3 can be activated by a wide range of activators including ATP and nigericin (Mariathasan et al., 2006). It is thought that ATP acts on its receptor P2X7 adrenergic receptor, leading to the opening of potassium ion channel and the efflux of K^+^. The efflux of K^+^ in turn triggers the assembly of NLRP3 inflammasome; being different from ATP, nigericin is a potassium ion carrier and can insert into the plasma membrane to form potassium channels which cause the efflux of K^+^, thus activating the NLRP3 inflammasome (Munoz-Planillo et al., 2013; He et al., 2016). We found that scutellarin inhibited NLRP3 inflammasome activation by both ATP and nigericin, suggesting that its action may on the downstream components of P2X7 receptor. Supporting this idea, our study showed that scutellarin markedly suppressed ASC speck formation and oligomerization upon NLRP3 activation by ATP or nigericin, suggesting their interference of NLRP3’s capacity in recruiting ASC to form inflammasomes.

One major concern is how scutellarin suppressed NLRP3’s capacity to assemble inflammasomes upon ATP or nigericin stimulation. Recent studies have demonstrated that NLRP3 activation has been negatively regulated by PKA signaling (Kim et al., 2017), which can be augmented by small molecules, such as bile acids (Guo et al., 2016), prostaglandin E2 (Mortimer et al., 2016), and dopamine (Yan et al., 2015), through their respective receptors; Ser/Thr phosphorylation of NLRP3 on PKA-specific sites prevents it from assembling inflammasomes. In light of the fact that scutellarin can increase cAMP levels (Tian et al., 2016) (and therefore increases the PKA activity), we proposed that scutellarin might block NLRP3 activation by enhancing PKA signaling. In support of this notion, selective PKA inhibitor H89 completely abrogated the inhibitory effects of scutellarin on pyroptosis in macrophages. H89 also augmented ATP-induced formation of ASC specks and entirely reversed scutellarin-mediated suppression of ASC specks. Accompanying this, scutellarin-induced phosphorylation of NLRP3 on PKA-specific sites was also blocked by H89 pretreatment. Besides, MDL12330A, an inhibitor of adenyl cyclase (AC) upstream of PKA signaling, also drastically abrogated scutellarin’s inhibitory effects on NLRP3 activation and pyroptosis. Altogether, these data indicated that scutellarin inhibited NLRP3 inflammasome activation and pyroptosis in macrophages by augmenting PKA signaling.

The formation of NLRP3 inflammasome provides a platform for the activation of caspase-1, which in turn converts pro-IL-1β into mature IL-1β. The release of mature IL-1β has been shown to be dependent on gasdermin D-mediated pyroptosis (He et al., 2015; Shi et al., 2015). Active caspase-1 leads to pyroptosis by cleaving gasdermin D to generate N-terminal product (gasdermin D-NT), which forms pores on the plasma membrane culminating in pyroptotic cell death (Chen et al., 2016; Ding et al., 2016; Liu et al., 2016; Sborgi et al., 2016). Consistent with these studies, our results showed that scutellarin not only inhibited caspase-1 activation and pyroptosis but also blocked mature IL-1β secretion into culture supernatants. Accompanying the suppression of mature IL-1β secretion, the release of other inflammatory intracellular components including HMGB1 had also been reduced. As HMGB1 is a critical damage-associated molecular pattern (DAMP) that is released into extracellular milieu during bacterial infections or cell damages triggering multiple inflammatory signaling (Lotze and Tracey, 2005; Yanai et al., 2012), the anti-inflammatory activities of scutellarin may be partly mediated by suppressing HMGB1 release during NLRP3-dependent pyroptosis. Thus, combined suppression of mature IL-1β release and DAMPs such as HMGB1 may account for the potent anti-inflammatory activities of scutellarin, highlighting its potency against NLRP3-mediated inflammation.

The activation of NLRP3 inflammasome has been suggested to play important roles in sepsis (Gong et al., 2015; Kim et al., 2016; Long et al., 2016; Hao et al., 2017; Lee et al., 2017), which is currently defined as life threatening organ dysfunction caused by a dysregulated host response to infection (Singer et al., 2016). In the early stages of sepsis, large amounts of proinflammatory cytokines including IL-1β, known as ‘cytokine storm,’ are produced leading to multiple organ injury and failure (van der Poll et al., 2017). Therefore, in the initial phase of sepsis, the anti-inflammatory therapy is effective and blocking the activity of IL-1β with biologic inhibitors has been used to prevent sepsis but showed limited effectiveness (Marshall, 2014). Recent studies proposed that blockade of NLRP3 activation, thus blocking the secretion of IL-1β and other inflammatory components, might have advantages over the use of biologic inhibitors of IL-1β. For example, small molecules, such as glyburide (Lamkanfi et al., 2009), MCC950 (Coll et al., 2015), and ketone metabolite β-hydroxybutyrate (Youm et al., 2015), have been shown to be inhibitors of NLRP3 inflammasome, thus being effective in treating NLRP3-related diseases. Consistent with these studies, we showed that oral administration of scutellarin significantly improved the survival of mice in bacterial sepsis. In line with the *in vitro* data, scutellarin administration also markedly decreased serum IL-1β levels and reduced infiltration of inflammatory cells in the liver. However, the bacterial burden in the peritoneal cavity was unchanged by scutellarin suggesting that it had no antibacterial activity, which is consistent with previous study showing no bactericidal activity for scutellarin (Lucarini et al., 2015). Thus, the improved survival of mice with bacterial sepsis was most likely due to attenuated systemic inflammation, as revealed by decreased serum IL-1β levels and reduced infiltration of inflammatory cells in the liver. These results suggest that scutellarin may be a potential therapeutic agent for the treatment of bacterial sepsis.

Previous studies had shown that 50 mg/kg and 100 mg/kg body weight of scutellarin (either intraperitoneally or orally administered) significantly attenuates inflammatory responses in a rat model of brain ischemia injury (Yuan et al., 2014; Wang W. et al., 2016). Another toxicity study indicated that oral administration of scutellarin at 500 mg/kg body weight daily for 30 days did not result in death or significant changes in hematology, blood chemistries or urinalysis, indicating that scutellarin is minimally toxic or non-toxic in rodents (Li et al., 2011). Based on these previous studies, we used 100 mg/kg and 200 mg/kg body weight of scutellarin for animal experiments. Instead of intraperitoneal injection, scutellarin was administered via oral gavage in order to avoid direct interference with bacteria in the peritoneal cavity. Our results showed that these two doses of scutellarin significantly reduced systemic inflammation of bacterial infected mice and increased their survival rates. These data indicate that oral administration of scutellarin may be an effective route conferring protective effects against systemic inflammation during bacterial infections.

## Conclusion

We demonstrated that scutellarin inhibited NLRP3 inflammasome activation and pyroptosis in macrophages through augmenting PKA signaling, unraveling a novel anti-inflammatory mechanism beyond previously identified inhibitory action on the NF-κB and Notch pathways (Tan et al., 2010; Yuan et al., 2014, 2015; Wang W. et al., 2016; Zhao et al., 2016). Furthermore, our data also suggest that scutellarin can be used to treat bacterial sepsis probably by blocking NLRP3 activation in the early stage of bacterial sepsis. As aberrant NLRP3 activation has also been implicated in more complex diseases including type 2 diabetes, gout, atherosclerosis and neurological diseases (Guo et al., 2015), scutellarin may have potential application in the treatment of such disorders. It is still unknown how scutellarin affects the PKA signaling pathway in macrophages. Although further research is warranted to elucidate the precise mechanism underlying this process, our data highlight the potential application of scutellarin for the treatment for NLRP3-related inflammatory diseases.

## Author Contributions

YL, Y-YJ, C-YZ, C-GL, and L-HX performed the cellular studies. C-GL, LY, W-JB, and Q-BZ performed the flow cytometry and immunofluorescence assays. Y-YJ and C-GL analyzed the data. X-HH and D-YO supervised the study. X-HH, D-YO, and YL wrote the paper.

## Conflict of Interest Statement

The authors declare that the research was conducted in the absence of any commercial or financial relationships that could be construed as a potential conflict of interest.
