# Supplementary material for: Scutellarin Suppresses NLRP3 Inflammasome Activation in Macrophages and Protects Mice against Bacterial Sepsis
Source: Front Pharmacol. 2018 Jan 9;8:975. doi: 10.3389/fphar.2017.00975 (PMC5767189; doi:10.3389/fphar.2017.00975)

Supplementary Materials

Scutellarin Suppresses NLRP3 Inflammasome Activation in Macrophages and Protects Mice against Bacterial Sepsis (Yi Liu, Yan-Yun Jing, Chen-Ying Zeng, Chen-Guang Li, Li-Hui Xu, Liang Yan, Wen-Jing Bai, Qing-Bing Zha, Dong-Yun Ouyang and Xian-Hui He)

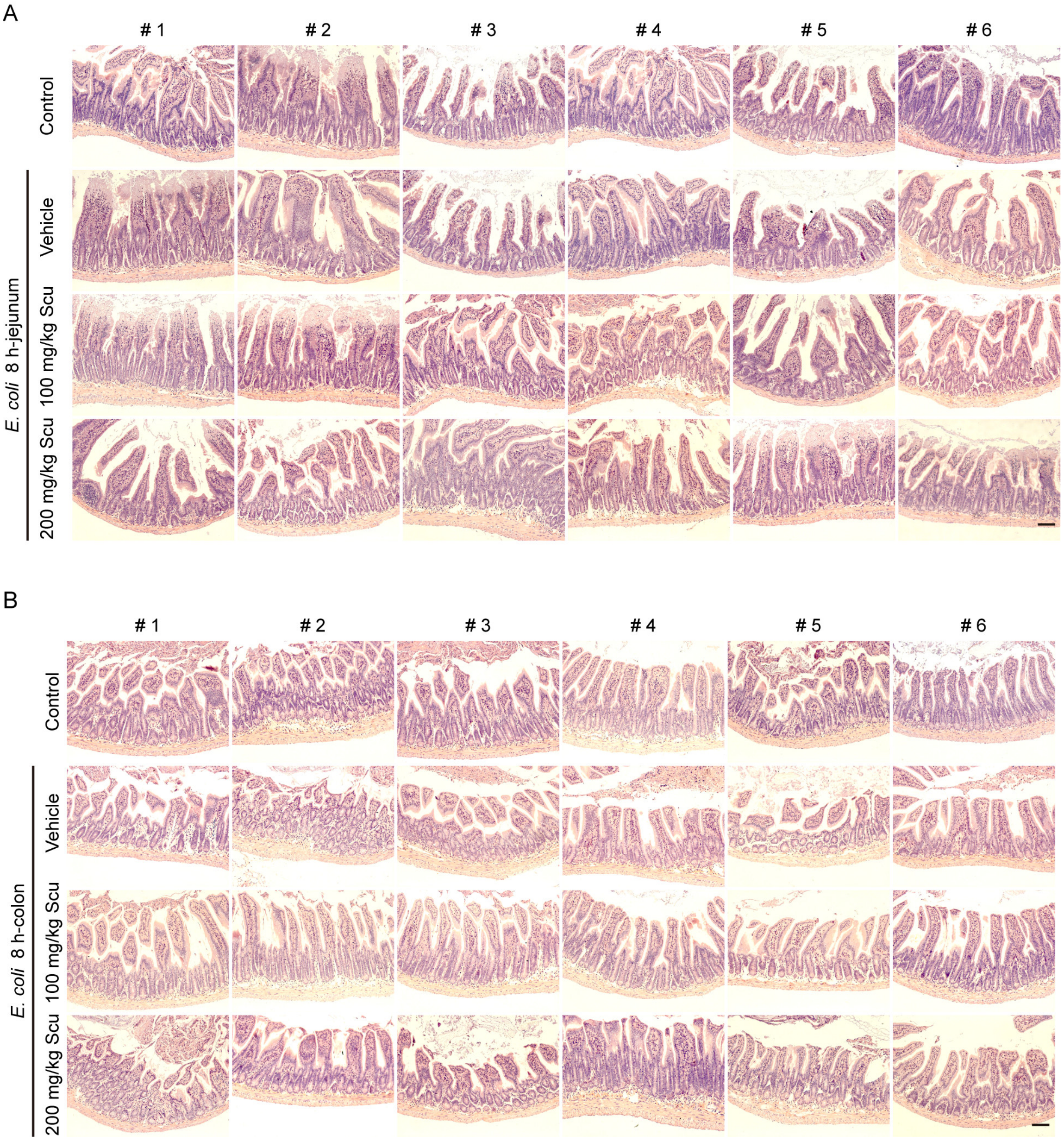

Figure S1 | Hematoxylin and eosin staining of the Jejunum and the colon of mice infected with *Escherichia coli*. C57BL/6 mice were administered once intragastrically (i.g.) with scutellarin (100 and 200 mg/kg body weight) or vehicle (2% Tween 80 in PBS) 3 h prior to bacterial infection (intraperitoneally) with viable *E. coli* (2.0×10^9 CFU/mouse). Mice were administered (i.g.) with scutellarin or vehicle once again at 1 h after the bacterial injection. Eight hours after bacterial infection, the intestines were isolated and fixed in 4% neutral formaldehyde solution. The sections in paraffin wax were stained by hematoxylin-eosin. Representative images from each mouse were presented. The numbers on the top indicate mouse number. Scale bar, 100 μm; Scu, scutellarin.

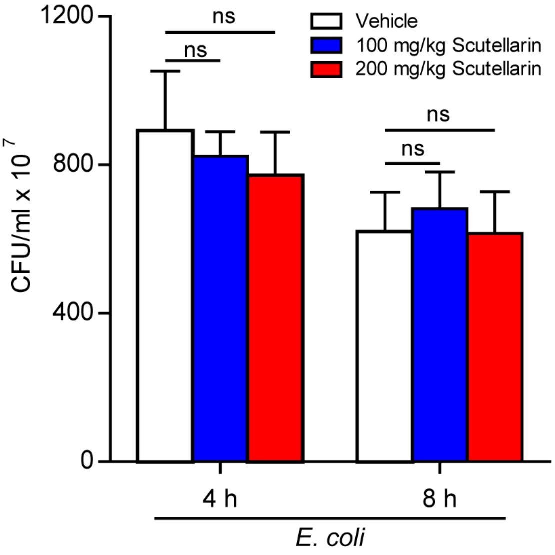

Figure S2 ︱ Scutellarin administration had minimal effects on the bacterial counts in the peritoneal cavity of mice. C67BL/6 mice were treated as indicated in **Figure S1**. The mice were sacrificed and peritoneal lavage fluids were collected with 1.5 ml PBS. Bacterial density was measured by using an ultraviolet-visible spectrophotometer (NanoDrop2000, Thermo Scientific), and the corresponding colony-forming units (CFUs) were determined on Luria Broth (LB) media agar plates. ns, not significant.

Western blotting source data:

Figure 1A

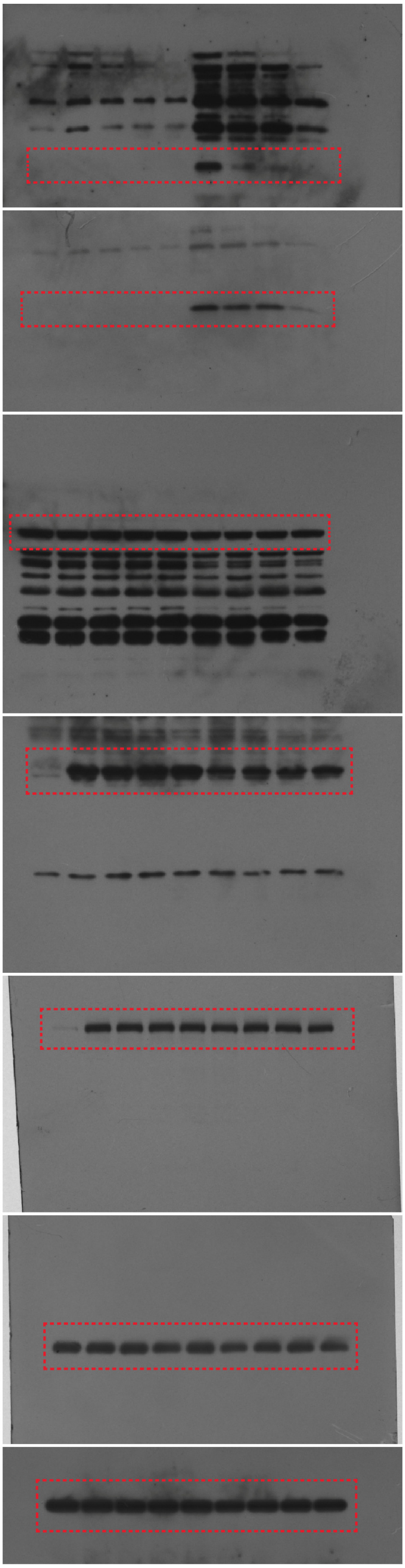

Figure 2A

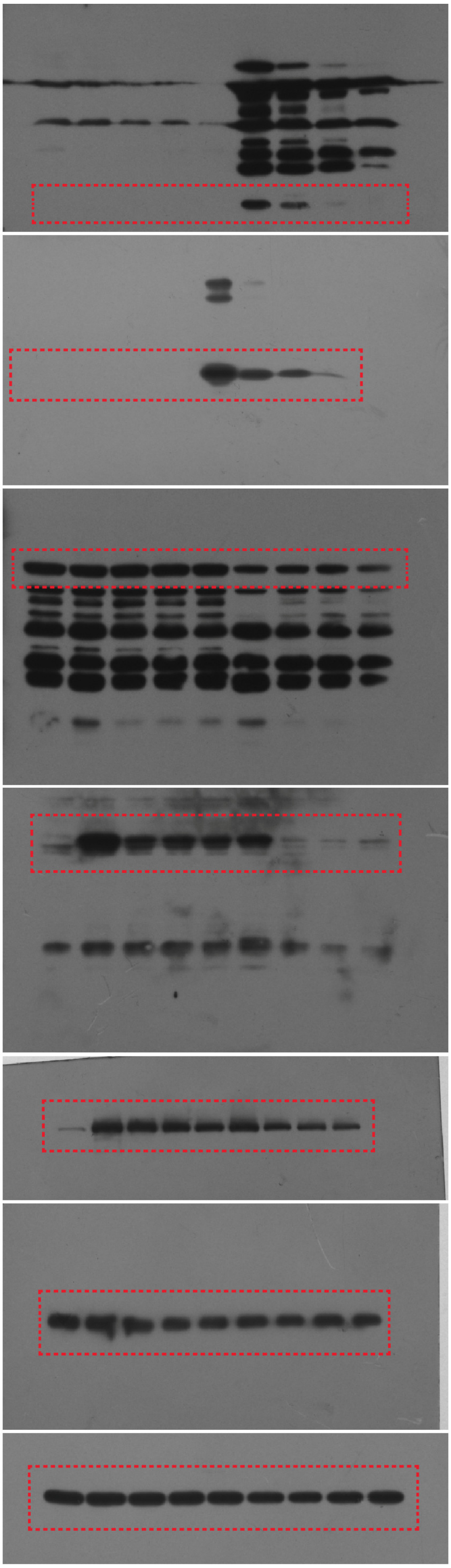

Figure 3C

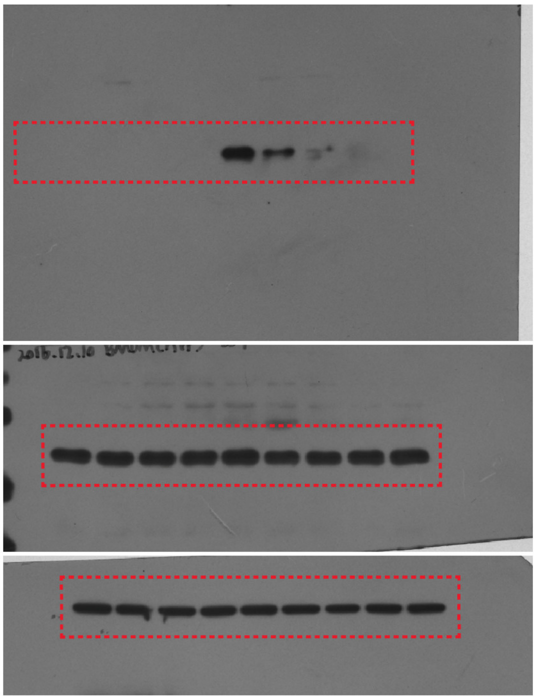

Figure 4C

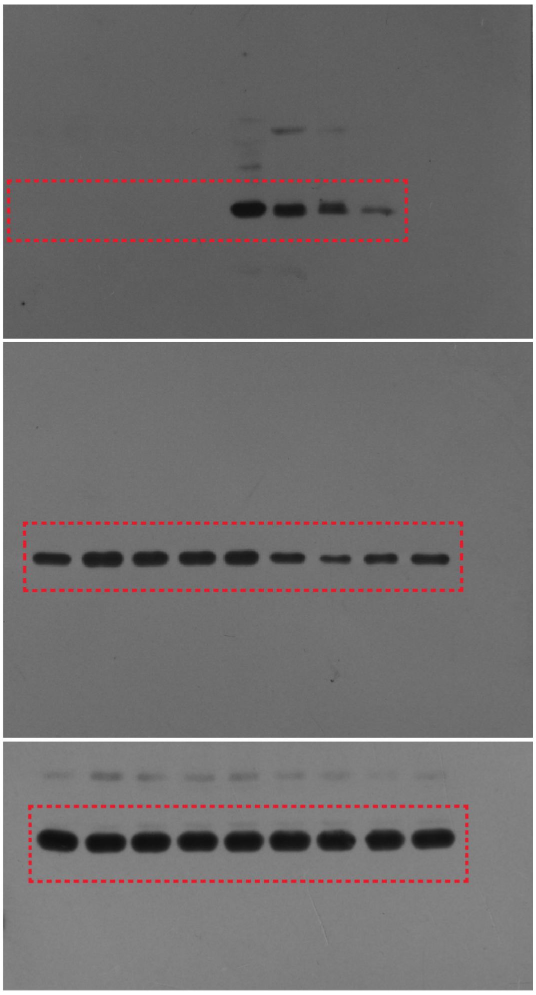

Figure 5C

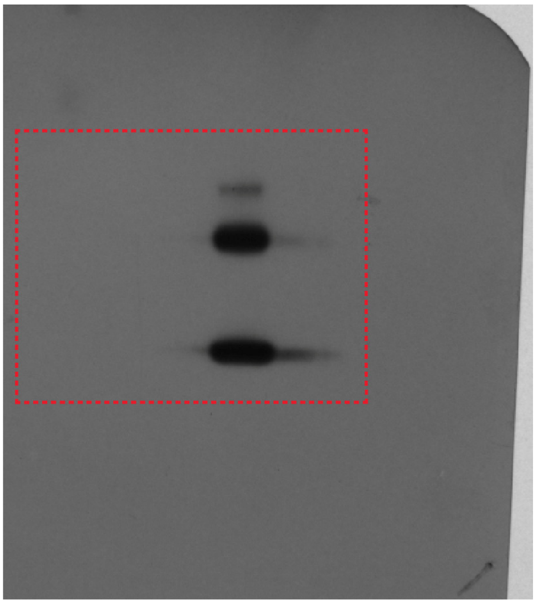

Figure 6C

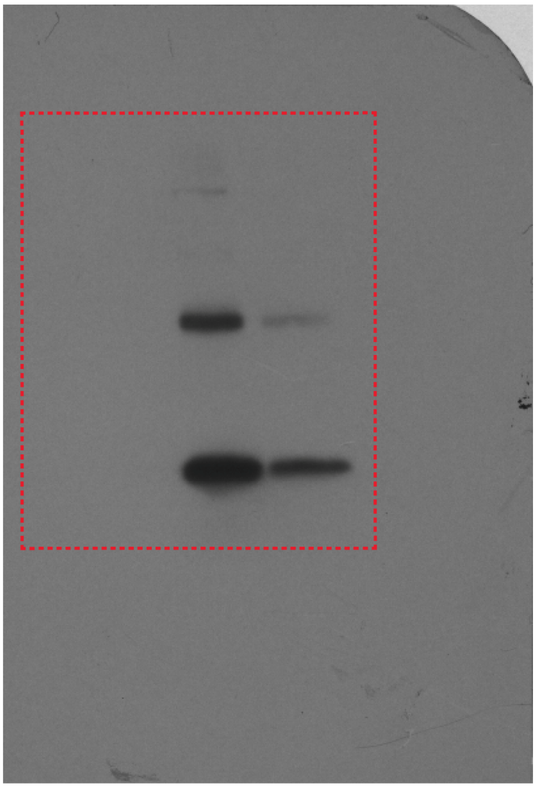

Figure 7A

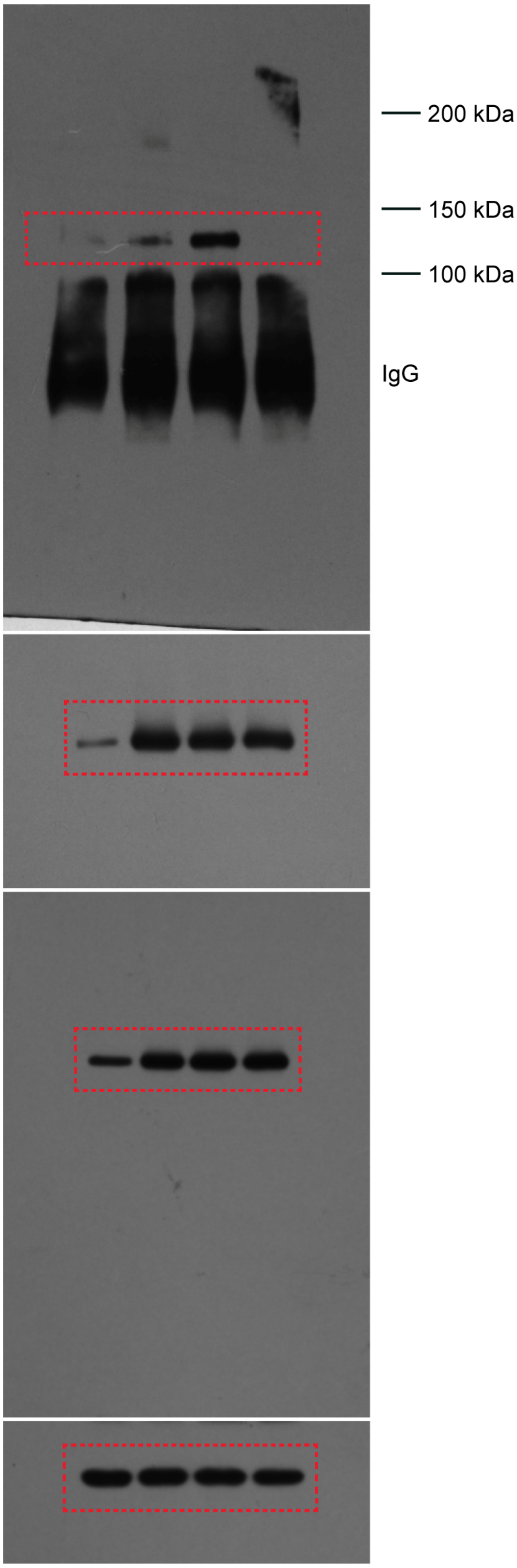

Figure 9C

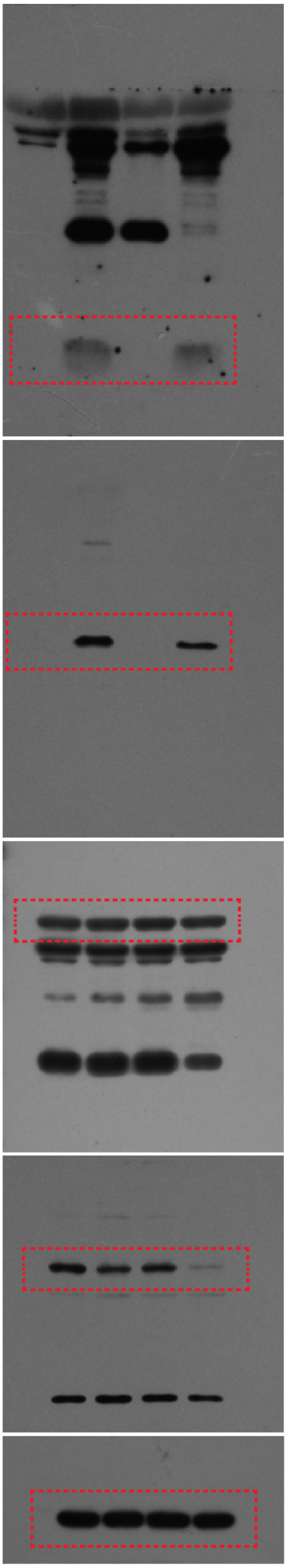

Supplement: Supplementary file 1 [file Data_Sheet_1.PDF]
